# X-chromosome tiling path array detection of copy number variants in patients with chromosome X-linked mental retardation

**DOI:** 10.1186/1471-2164-8-443

**Published:** 2007-11-29

**Authors:** I Madrigal, L Rodríguez-Revenga, L Armengol, E González, B Rodriguez, C Badenas, A Sánchez, F Martínez, M Guitart, I Fernández, JA Arranz, MI Tejada, LA Pérez-Jurado, X Estivill, M Milà

**Affiliations:** 1 Biochemistry and Molecular Genetics Department, Hospital Clínic and IDIBAPS (Institut d'Investigacions Biomèdiques August Pi i Sunyer), Barcelona, Spain; 2 Genes and Disease Program. Center for Genomic Regulation (CRG-UPF), Barcelona, Spain; 3 Microarray Laboratory, Bioinformatics and Genomics Program, (CRG-UPF), Barcelona, Spain; 4 Genetics Unit, Universitat Pompeu Fabra, Program in Molecular Medicine and Genetics, Hospital Vall d'Hebron, Barcelona, Spain; 5 Unidad de Genética. Hospital Universitario La Fe. Valencia, Spain; 6 Laboratori Genètica, UDIAT-Centre Diagnòstic, Corporació Sanitaria Parc Taulí, Institut Universitari Parc Taulí-UAB, Sabadell, Spain; 7 Instituto de Biología y Genética Molecular (IBGM). Universidad de Valladolid, Spain; 8 Unitat de Metabolopaties, Hospital Universitari Materno-Infantil Vall d'Hebron, Spain; 9 Molecular Genetics Laboratory, Cruces Hospital, Barakaldo-Bizkaia, Spain; 10 GIRMOGEN (Spanish Network for Mental Retardation), Spain; 11 Centre for Biomedical Research on Rare Diseases (CIBERER), ISCIII, Barcelona, Spain

## Abstract

**Background:**

Aproximately 5–10% of cases of mental retardation in males are due to copy number variations (CNV) on the X chromosome. Novel technologies, such as array comparative genomic hybridization (aCGH), may help to uncover cryptic rearrangements in X-linked mental retardation (XLMR) patients. We have constructed an X-chromosome tiling path array using bacterial artificial chromosomes (BACs) and validated it using samples with cytogenetically defined copy number changes. We have studied 54 patients with idiopathic mental retardation and 20 controls subjects.

**Results:**

Known genomic aberrations were reliably detected on the array and eight novel submicroscopic imbalances, likely causative for the mental retardation (MR) phenotype, were detected. Putatively pathogenic rearrangements included three deletions and five duplications (ranging between 82 kb to one Mb), all but two affecting genes previously known to be responsible for XLMR. Additionally, we describe different CNV regions with significant different frequencies in XLMR and control subjects (44% vs. 20%).

**Conclusion:**

This tiling path array of the human X chromosome has proven successful for the detection and characterization of known rearrangements and novel CNVs in XLMR patients.

## Background

Mental Retardation (MR) is a common disorder affecting 1–3% of the general population [1]. An excess of affected males over females has been noted among mentally delayed patients, especially in moderate to severe MR. This phenomenon has usually been explained by the presence of many genes responsible for MR on the X chromosome. X-linked MR (XLMR) is an heterogeneous condition representing an important proportion of patients affected by MR and can be classified either as nonsyndromic, when mental delay is the only symptom, or as syndromic when MR is associated with other specific clinical features.

Copy number variations (CNVs) are defined as copy number changes including deletions, insertions and duplications of genomic regions that range from one kilobase (kb) to megabases (Mb) in size. CNVs can influence gene expression by directly disrupting genes or by altering gene dosage [2,3], and some are involved in specific genetic disorders such as microdeletion and microduplication syndromes (e.g. Williams-Beuren, Smith-Magenis or DiGeorge syndromes). Several studies report an incidence of cryptic chromosomal imbalances in about 10–25% of MR cases [4-6]. Other copy number variations are present as polymorphisms in the general population without apparent relation to disease [7-10]. Several of these cryptic chromosomal rearrangements occur in regions flanked by segmental duplications or low-copy repeats and likely result from non-allelic homologous recombination between different copies of these repeats [11].

Nowadays, array-based comparative genomic hybridization (aCGH) represents a useful and cost-effective tool for the detection of submicroscopic copy number changes in genetic diseases [12]. Here, we describe the development, validation and use of a BAC derived tiling path array covering the entire euchromatic portion of the human X chromosome, which has allowed the screening for copy number changes in 54 XLMR patients.

## Results

### Validation of the X-chromosome BAC array

Sensitivity and specificity of the X-array to detect copy number changes were tested with DNA from four patients with known cytogenetic aberrations on the X chromosome (see material and methods). In all these individuals, the corresponding changes in copy number were clearly detected and confirmed by the array-CGH. A series of sex mismatched hybridizations of controls versus controls, as well as self-self hybridizations allowed the detection of clones in the array that performed abnormally. Criteria for considering problematic clones were: 1) clones with absolute value of log_2 _ratios > 0.2 in self-self hybridizations, 2) clones with high standard deviations (SD) (> 2 times the deviation of deviations) in different hybridizations and 3) clones displaying normal log2 ratios within a known aberration. Thirty clones were considered problematic and were excluded from further analyses.

### Array CGH findings in X-linked MR patients

**Figure 1:**
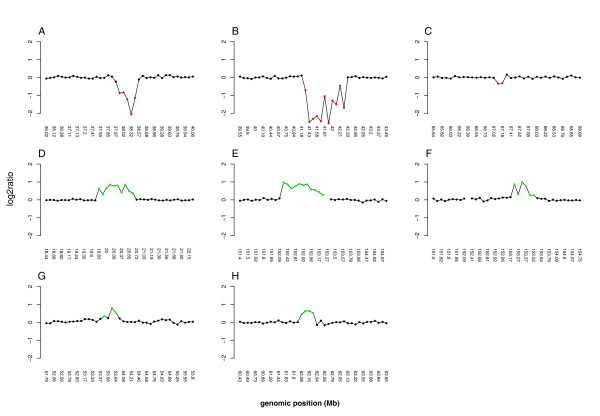
Array CGH profiles for XLMR patients with relevant CNVs. Each dot represents the mean log2 transformed and Lowess normalized test over reference intensity ratio (y-axis), which is derived from four independent replicate spots on the array, in a replicate dye swap experiment. The Mb position of the clones on the X chromosome is displayed in the x-axis, ordered from p-telomere to q-telomere on the basis of physical mapping positions, according to the hg17 assembly of the UCSC genome browser [34]. (A) 400-kb deletion at Xp11.4 (37.9–38.8 Mb) in patient 1. (B) Deletion spanning a region of 1 Mb (43.01–43.98 Mb) in Xp11.3 in patient 2. (C) Deletion of two clones in Xq12 spanning 82 kb (67.10–67.18 Mb) in patient 3. (D) 800-kb duplication at Xp22.12 (19.82–20.65 Mb) in patient 4. (E) Duplication of 700 kb at Xq28 (152.4–153.1 Mb) in patient 5. (F) Duplication of 250 kb at Xq28 (153.2–153.5 Mb) in patient 6. (G) Duplication of 400 kb at Xp11.22 (53.53–53.94 Mb) in patient 7. (H) Duplication of 180 kb at Xq12 (62.07–62.26 Mb) in patient 8.

Fifty-two patients with unexplained XLMR and two patients with an X-linked trait were analyzed using the tiling path X chromosome array. Copy number variations were observed in 26 patients (48%) and in 8 patients (14.8%), we identified imbalances probably causative of the phenotype observed in the patients. An overview of these imbalances is shown in Figure 1. Table 1 summarizes the phenotype and genotype of these 8 patients.

**Table 1:** Patients with mental retardation and imbalances detected with the X-chromosome tiling path BAC array not identified in control samples

**Case**	**Region**	**Gain/loss**	**Size kb**	**Origin**	**Phenotype**	**Altered clones**	**Affected genes**	**Confirmatory studies**
Known								
1	Xp11.4	Loss	400	Inherited	OTC deficiency, neurological deterioration	RP13-486L5, RP11-401A21, RP11-727P7, CTD-2135N1, RP11-416I6, RP11-604A4	RPGR, **OTC**, **TMSF4 **(TSPAN7), SRPX	*Arranz et al*., 2007
2	Xp11.3	Loss	1Mb	Inherited	Norrie disease, severe psychomotor retardation, epilepsy, microcephaly,	RP11-558I17, RP11-561O22, RP11-643D7, RP11-455O7, RP11-72J3, RP11-291H16, RP11-355O20, RP11-11B9, RP11-634A3, RP11-110B2, RP11-605I23	**NDP**, MAOA, MAOB, EFHC2	Rodriguez-Revenga *et al*., 2007
Causal								
3	Xq12	Loss	82	Inherited	Severe MR, strabismus, cerebellar hypoplasia, Dandy-Walker malformation, prominent chin, high nasal root	RP11-586C9, RP11-466E18	**OPHN1**	MLPA PCR
4	Xp22.12	Gain	800	Inherited	Mild MR, Robust built, dyslexia, facial dysmorphism	RP11-191B9, RP11-393H10, RP11-382L18, RP11-254G9, RP11-367L17, RP11-203E14, RP11-703P16, RP11-48D14, RP11-712B24, RP11-158M19	**RPS6KA3**, CXorf23, EIF1AX	MLPA
5	Xq28	Gain	700	Inherited	Severe MR, psychomotor retardation, hypotonia,	RP11-54I20, RP11-437K1, RP11-314B3, RP11-398P14, RP11-846A22, RP11-617G6, RP11-485N14, RP11-119A22, RP11-333O6, RP11-330B2, CTD-2238E23	**SLC6A8**, PLXNB3, L1CAM, **MECP2**, IRAK1, ARHGAP4, OPN1MW, CXorf2, TKTL1, PDZK4, ABCD1, PNCK, DUSP9, FAM58A, ATP2B3	MLPA
6	Xq28	Gain	250	Inherited	Moderate MR, facial dysmorphism, microcephaly,	RP11-666B23, RP11-316P8, CTD-2511C7, CTD-2242O14, RP11-696D6, RP11-103M23	**GDI1**, FLNA EMD, TAZ, TKTL1, RPL10, DNASE1L1	MLPA RT-PCR
Causal?								
7	Xp11.22	Gain	420	Inherited	Moderate MR	RP11-390E18, RP11-585D6, RP11-555J12, RP11-272G22, RP13-444K19	HUWE1, **PHF8?**	MLPA
8	Xq12	Gain	350	ND	Moderate MR	RP11-414C19, RP13-547B18, RP11-655E9, RP11-715J23	EDA2R	MLPA

ND, not determined; in bold are represented the genes related to MR.

### Known genomic aberrations

In the two patients with a presumed rearrangement in the short arm of the X chromosome (Xp11.4 and Xp11.3), aberrations in these regions were identified by the array CGH.

#### Case 1

Case one was a newborn biochemically diagnosed of OTC deficiency. His mother had been diagnosed of OTC deficiency and associated neurological impairment since childhood. We identified a small deletion (400 kb) at Xp11.4 both in the patient and his mother. This deletion involved six adjacent clones (RP13-486L5, RP11-401A21. RP11-727P7, CTD-213N1, RP11-416I6 and RP11-604A4) and *OTC*, *TSPAN7 *(*TM4SF2*) and *RPGR *genes at Xp11.4 [13]. Deletion of the *OTC *and *RPGR *genes in the patient was confirmed by PCR. Deletion of the *TMSF4 *gene was confirmed by MLPA both in the patient and his mother (see Additional file 1A).

#### Case 2

Case two was a boy with clinical suspicion of Norrie disease. He presented a severe psychomotor retardation without verbal language skills, microcephaly, bilateral retinal detachment and epilepsy. A deletion spanning 11 clones with an estimate size of 1 Mb was identified at Xp11.3 in the patient and his mother (see Additional file 1B). Array results confirmed the clinical suspicion of Norrie disease. The deletion involves *NDP, MAOA, MAOB, EFHC2 *genes. In this case, epilepsy, not a common trait of Norrie disease, is probably caused by the deletion of the *EFHC2 *gene [14].

### Patients with non-syndromic mental retardation

In 6 out of 52 patients, we identified genomic imbalances in regions, which included at least one gene related with MR (Table 1). Family studies supported the causal relationship with the MR phenotype observed in the index cases and demonstrated that the imbalance was inherited from an obligate carrier, except in case 8.

#### Case 3

Case three was one of four MR siblings from healthy, consanguineous parents (see Additional file 1C). The major clinical features were mild mental retardation, strabismus, hypogenitalism, a marked synophrys or medial eyebrow flare, a prominent nose and a broad high nasal root. aCGH detected a deletion spanning two clones on Xq12 affecting *OPHN1 *gene (Figure 1C). MLPA detected a deletion of exon 21 of this gene. The estimation of the altered size was achieved by PCR which revealed a deletion involving only exons 21 and 22. The deletion fully segregates with the phenotype in the family. X-inactivation analysis showed a skewed X-inactivation pattern in the carrier mother (99/1) and not skewed in the carrier sister (72/28).

#### Case 4

Case 4 was a boy affected by mild MR, dyslexia and mild dysmorphic features. A duplication of approximately 1 Mb was detected at Xp22.12 (Figure 1D), affecting four genes (*CXorf23*, *MAP7D2*, *EIF1AX *and *RPS6KA3*). MLPA study detected duplication of *RPS6KA3 *in the patient, one sister, mother and affected uncles (see Additional file 1D). X-inactivation studies showed a random X-inactivation pattern in the mother (41/59).

#### Case 5

This boy was born to healthy nonrelated parents at term. His psychomotor development was delayed with severe MR, generalized hypotonia, limited walking and speech delay. He has a family history of MR (affected relatives not studied)(see Additional file 1E). He presented some mild facial dysmorphisms such as narrow forehead, thin nose, small mouth and marked dark rings under the eyes. aCGH showed a duplication of at least 700 kb involving 15 genes at Xq28 (Figure 1E), including *SCL6A8 *and *MECP2 *genes. Specific MLPA for this region confirmed duplication of *MECP2*, *L1CAM *and *IRAK1 *genes in the patient and his mother.

#### Case 6

This boy was diagnosed of mild MR with an IQ of 58. He was born at 8 months from non-consanguineous parents after a pregnancy affected by acute pancreatitis. He presented neonatal seizures and cyanotic crisis. He acquired sedestation at 6 months, deambulation at 14 months and speech at 15 months. He showed psychomotor delay and learning disabilities, microcephaly, hyperkinesias and other mild dysmorphic features such as medial eyebrow flare and deep palate. A duplication spaning 6 clones with an estimate size of 250 kb was identified at Xq28 (Figure 1F). MLPA confirmed duplication of *GDI1*, *FLNA *and *EMD *genes and discarded any alteration in the *MECP2 *gene in the patient, two affected relatives and two carrier women (see Additional File 1F). RT-PCR studies revealed a significantly higher *GDI1 *expression in the patient and his affected uncle (from 2.8 to 5 fold compared to four male controls; (SD = 0.19 Ct controls; SD = 0.09 Ct patient; SD = 0.25 Ct affected uncle). The carrier mother showed a complete skewed X-inactivation (100/0) inactivating the X-chromosome inherited by the patient.

#### Case 7

Case 7 was a boy affected by moderate MR, and minor dysmorphic features. aCGH detected a duplication of approximately 420 kb spanning the *HUWE1 *gene and probably the *PHF8 *gene at Xp11.22 (Figure 1G). The development of MLPA custom probes for *HUWE1 *and *PHF8 *allowed confirmation of the duplication of *HUWE1 *gene both in the patient and his affected brother (see Additional file 1G) and discard implication of *PHF8 *gene in the duplication. His mother was carrier of the duplication. X-inactivation pattern was skewed in the mother (90/10) and not skewed in the aunt (77/23). The screening of 75 normal male controls did not detect this CNV in any of them.

#### Case 8

Case 8 was a boy with moderate mental retardation and minor facial dysmorfisms. He has a family history of MR although no family members were available for study (see Additional File 1H). A duplication of approximately 350kb, involving four clones on Xq12 was identified by aCGH (Figure 1H). The duplication spans the *EDA2R *gene that encodes a receptor of the tumor necrosis factor receptor family. MLPA screening with custom probes for *EDA2R *gene confirmed the duplication in the patient and discarded it in 75 male controls.

Some other variations identified in XLMR patients correspond to previously reported CNVs present in the reference database of genomic variants [15]. The most common ones were deletions and reciprocal duplications in the Xq28 and Xq26.3 regions of an average size of 200 kb and 140 kb, respectively. The proportion of patients carrying CNVs was double than that of controls (44% vs. 20%). Further description of the identified CNVs is provided in Table 2. In general XLMR patients presented more changes and duplications were more common in XLMR patients than in controls (Table 3).

**Table 2:** X-chromosome CNVRs detected in patients and controls using the X-chromosome tiling path BAC array.

Region	Controls (20)	Cases (54)	Start	Clone	End	Clone	Size kb	Gain/Loss	OMIM Genes within CNVs
Xp22.33	-	1	2497952	RP11-325D5	2773932	RP11-457M7	278	Gain	CD99, XG, GYG2
Xp22.31	1	-	9304013	RP11-951B16	9510840	RP11-29K8	207	Gain	TBL1X
Xp21.2	1	-	30597301	RP11-710J8	30681589	RP11-642K22	84	Loss	TAB3
Xp11.23	-	1/1	47732169	RP11-423H3	47806532	RP11-38O23	74	Loss/Gain	SSX5, SSX6, LOC389852
Xq26.3	-/1	3/4	134591734	CTD-2225C20	134733742	RP11-111C16	142	Loss/Gain	SAGE1, MGC88118, MGC27005
Xq28*	1/-	7/7	152963046	CTD-2149G5	153072850	RP11-330B2	110-315	Loss/Gain	OPN1LW, OPN1MW
Xq28	1	-	151551673	RP11-793M20	151886266	CTD-2515E20	335	Loss	MAGEA6, MAGEA12, MAGEA2B, MAGEA2, MAGEA2B, CSAG2, MAGEA3

**Table 3:** Summary of changes detected both in controls and XLMR patients

		**Controls (20)**	**Patients (54)**
**Samples with CNVs**		20% (4/20)	48% (26/54)
**Total of CNVs**		5	32
**Dups**		20% (1/5)	56,2% (18/32)
**Dels**		80% (4/5)	43,7% (14/32)
**CNVs/sample**	1CNV	75% (3/4)	71,9% (23/32)
	2CNVs	25% (1/4)	28,1% (9/32)
**Size**		100–400kb	100–900Kb

## Discussion

The applicability of the array-based comparative genomic hybridization technology to detect copy number changes in mentally delayed patients has been described in a number of previous reports. An incidence of cryptic imbalances in about 10–25% of the cases has been reported [4,5,16-18]. With the aim of identifying novel aberrations involved in the cognitive impairment in XLMR patients, we have developed a tiling path X chromosome array for CGH with a 100 kb resolution. Clinically relevant imbalances were identified in 8 cases (14,8%) with genomic sizes ranging between 100 kb and 900 kb. In all these cases we have identified genes related to MR that could be responsible for the phenotype in these patients (Table 1). In cases 1 and 2, array CGH supported the previous clinical suspicion and helped to roughly delineate the location of breakpoints for the different aberrations. In case 3, the identification of an 82 kb deletion, affecting exons 21 and 22 of the *OPHN1 *gene at Xq12, demonstrated the usefulness and accuracy of array CGH to detect small copy number changes. In cases 4, 5 and 6 we identified duplication of *RPS6KA3, MECP2*, and *GDI1 *genes, respectively, all of them implicated in mental retardation and neurological disease. Mutations in *RPS6KA3 *are responsible for the Coffin-Lowry Syndrome and *GDI1 *has been related to nonsyndromic MR forms. As previously pointed out [19] we propose that the copy number alteration of dosage sensitive genes *RPS6KA3 *and *GDI1 *may be the major cause for the mental retardation in cases 4 and 6, respectively. In fact, case 6 and related family members carrying the duplication at Xq28 presented higher GDI1 mRNA levels than controls. As far as we are aware, there is only other report describing duplications of *RPS6KA3 *and *GDI1 *genes causing MR. Froyen *et al*.,(2007) described a boy with psychomotor delay with a small duplication of 0.3Mb in size involving the XLMR genes *FLNA *and *GDI1*, among others [20]. In the same work, they also detected a duplication of 21 Mb involving known MRX genes such as *RPS6KA3, CDKL5 *and *NLGN4X *in a patient affected by severe psychomotor delay. The detection in our series of two cases with smaller duplications involving *GDI1 *and *RPS6KA3 *genes reinforces the idea that increased gene dosage of these genes may be related to abnormal cognitive impairment.

In cases 7 and 8 we detected duplications of regions in which no genes involved in MR have been described. In case 7, the duplication at Xp11.22 was proximal but close to the MR related *PHF8 *gene [21]. MLPA only confirmed duplication of *HUWE1 *gene and although *PHF8 *is not implicated in the duplication, it might be somehow influenced by this CNV. In case 8, the duplication at Xq12 was confirmed by MLPA of *EDA2R *gene, which encodes a tumor necrosis factor receptor. Although the imbalance was confirmed by other molecular method, no DNA from the parents was available for genotyping and we could not establish the causativeness of the aberration in the observed phenotype. Nevertheless, duplications of *HUWE1 *and *EDA2R *have not been detected upon screening 75 male controls neither have ever been reported as genomic polymorphisms, suggesting that these changes are not very common in the general population. Nevertheless, further studies are needed in order to clarify their role in MR.

It has been shown that in several X-linked disorders, the X-inactivation process evidences a bias in mothers of affected individuals [22]. We have investigated the X-inactivation pattern in six female carriers. Fifty per cent of them showed a skewed X-inactivation of the chromosome carrying the mutant allele, which is in agreement with the literature [22]. However we have to bear in mind that this inactivation pattern has been observed in blood samples and we do not know what is happening in other tissues.

Until now among mentally retarded patients, deletions were the most commonly genomic aberrations identified. Recently, the use of array technologies has led to the detection of new duplications in mentally retarded patients and the description of new syndromes, i.e. *MECP2 *duplication syndrome [23]. The identification of new cases harboring duplications in these genes should be of help in order to elucidate their potential involvement in XLMR.

The number of described CNVs in human genome is exponentially increasing due to the high number of genome wide analyses. Nowadays there are reported more than 3500 CNVs, 110 on the X-chromosome [15]. We described here seven different CNV regions in the X-chromosome (Table 2). All these CNV regions overlap with others previously identified [10,24,25], and all are associated with segmental duplications. One of the most common CNV both in XLMR patients and in control subjects is at Xq28, at approximately two Mb from the telomere and associated to known segmental duplications. CNV regions and segmental duplications are not uniformly distributed throughout the genome, being significantly overrepresented in number within two Mb of telomeres and centromeres [26]. This Xq28 polymorphic region encompasses the opsin cluster, a family of genes involved in color perception. It has been previously reported that many genes involved in the senses such as olfactory receptors and opsins (cone pigments) associate with CNVs [10,27]. The second most common CNV in XLMR patients is at Xq26.3. This region is polymorphic in the general population [10,25,28] and it contains several cancer-related genes such as SAGE and MAGE tumor antigen families. Curiously, CNVs are more common in XLMR patients than in controls; i.e. 26% of patients were polymorphic for the Xq28 region vs. 5% of controls. Furthermore, the percentage of CNVs is statistically higher in XLMR than in controls (proportion test Z = 2,51, p = 0,012; Fisher's exact test: P-Value = 0,035). Also the average size is higher of CNVs seems to be higher in patients than in controls (Table 3). We still do not know the significance of some of these CNVs, i.e. they can influence expression of other genes. Further characterization of these variable regions, including quantitative analyses, opens a new field of study that should assist to understand the role of this genomic variation in mental retardation.

## Conclusion

The X chromosome aCGH presented here has been proven successful for the detection of novel CNVs and characterization of known rearrangements in XLMR patients. Even more we have detected some polymorphic CNV that seem to be more frequent in XLMR than in controls. Further characterization of these variable regions, including quantitative analyses, opens a new field of study that should assist to understand the role of this genomic variation in MR.

## Methods

### Patient and control samples

We studied a total of 54 unrelated patients with mental retardation: 52 MR patients belonging to families compatible with an X-linked inherited MR and two patients with suspicion of an X chromosome deletion due to their clinical manifestations (*OTC *deficiency and Norrie disease). All XLMR patients displayed normal karyotype, and CGG-expansions of the *FMR1 *gene were ruled out. We also studied four samples with cytogenetically visible copy number aberrations validated by molecular cytogenetic techniques (46, XX, dupXq22-q26; 46, XX, dupXq28; 46, XX, delXq27-qter; 46, XX, dupXp11-p21) and a series of 20 control males.

This study has been approval by the ethic committee of the Hospital Clinic of Barcelona. All the subjects provided written informed consent for the use of their phenotypic and genetic data.

### Construction of the tiling-path X chromosome CGH array

The X chromosome-specific tiling path array consists of about 1,600 genomic BAC clones derived from the human X chromosome plus 3 Drosophila negative control clones. The clone set used to produce this array was mainly derived from the 32 K human BAC library from the Children Hospital Oakland Research Institute [29]. Gaps were covered using BACs from other libraries (mainly RP11). Slides contained quadruplicates of the 1,600 clones providing an average density of at least one clone per 100 kb along the entire euchromatic portion of the X chromosome. The production of the X-array, probe preparation, and hybridization on the array were performed in the Microarray Unit of the Center for Genomic Regulation (CRG, Barcelona, Spain). BAC DNA was isolated from 1.5 ml bacterial cultures using the Montage BAC96 Miniprep kit following manufacturer's instructions (Millipore, Billerica, MA). DNA amplification by DOP-PCR was done as previously described [30]. PCR products were purified using the Montage PCR_96 _Plates kit (Millipore, Billerica, MA) and quantified using the PicoGreen dsDNA Quantification kit (Invitrogen, Life technologies, Carlsbad, CA). Purified products were dried, dissolved at 400 ng/μl in 50% DMSO and spotted in quadruplicate using a VersArray ChipWriter™ Pro System (Bio-Rad).

### Sample hybridization

Hybridization was performed as previously described [31]. For each hybridization, 400 ng of test and control DNA were labeled by random priming using the BioPrime Array CGH Genomic Labeling System (Invitrogen, Life technologies, Carlsbad, CA). Reversed-dye labeling of the samples was always used to minimize the effect of dye-specific artifacts. Each patient was hybridized against a sex-matched pool of 50 healthy controls. Arrays were scanned using an Agilent G2565BA MicroArrayScanner System (Agilent Inc., Palo Alto, CA) and the acquired images were analyzed using GenePix Pro 6.0 software (Axon, Molecular Devices) using the irregular feature finding option. Extracted raw data was filtered and Lowess normalized using Bacanal (Lozano *et al*., unpublished), an in-house developed suite for microarrays analysis linked to a management system.

On top of an Apache server and an Oracle database, a combination of different R packages process the raw data obtained from GenePix, performs a quality control of the signals, performs a loess normalisation of spot signals (using LIMMA package) taking into account spot quality control and background intensities and tries to identify copy number variable regions using circular binary segmentation (using the DNAcopy R package). SD of all X-chromosome clones was calculated for each hybridization experiment. Genomic imbalances were determined based on log2 of the Cy5/Cy3 ratios of the average of the four replicates, and regions were considered as duplicated or deleted when at least two consecutive clones exceeded the ± 0.2 range.

### Confirmatory analyses

Putative copy number aberrations were confirmed by other molecular techniques such as *Multiplex Ligation-dependent Probe Amplification *(MLPA), quantitative PCR (qPCR) and/or PCR

### Multiplex Ligation Probe Amplification

All 54 samples were included in a parallel MLPA screening [32] in which we used a commercial specific probe mixture with 43 probes for 14 known genes responsible for X-linked MR (Salsa P106, MRC-Holland, Amsterdam, The Netherlands). Additionally for *MECP2 *gene we used a specific kit for Rett syndrome that includes probes for *MECP2*, *L1CAM *and *IRAK1 *genes (Salsa P015C, MRC-Holland, Amsterdam, The Netherlands). The assays were performed following manufacturer's recommendations (MRC-Holland, Amsterdam, The Netherlands). Specific MLPA probes were designed for *HUWEI1*, *PHF8*, *EMD*, *EDA2R *and *FLNA *genes for screening of 75 male controls (see Additional file 2).

### PCR

PCR was always performed for confirmation of our array-CGH data when losses were detected. Primers for *OPHN1 *gene *(case 3) *were designed with the Primer3 software (primer3 V0.3.0) (see Additional file 3).

### Quantitative RT-PCR

Expression of *GDI1 *gene was analyzed by real time RT-PCR using TaqMan probes (ref. Hs00181741_m1, Applied Biosystems, CA, USA). Total RNA of the patient and other affected males in the family was extracted from whole blood. Relative quantification was performed against a control amplicon of the GUSB mRNA following manufacturer instructions (Applied Biosystems, CA, USA).

### X-inactivation

Androgen-receptor gene methylation assay to assess the methylation status was performed over lymphocyte genomic DNA of female carriers, as previously described [33]. Results are presented as the percentages of inactivation of both alleles. Skewed alleles were considered when the inactivation percentage was over 80%.

## Authors' contributions

IM carried out the extraction of DNA samples, construction and validation of the tiling-path X chromosome CGH array, analysis of array results, MLPA analysis, interpretation of array results and preparation of the manuscript ; LRR carried out the extraction of DNA samples, FISH experiments, PCR analysis and participated in the preparation of the manuscript; LA carried out design of the tiling-path X chromosome CGH array, interpretation of data, preparation of the manuscript and participated in the revision of the manuscript; EG carried out the construction and validation of the tiling-path X chromosome CGH array, hybridization of samples, interpretation of array results, revision of the manuscript; BR carried out the design of custom MLPA probes and participated in the revision of the manuscript; CB participated in the interpretation of data and revision of the manuscript; AS carried out the clinical evaluation of patients and revision of clinical data; FM carried out the collection of samples and acquisition of clinical data and participated in the revision of the manuscript; MG carried out the collection of samples and acquisition of clinical data participated in the revision of the manuscript; IF carried out collection of samples and acquisition of clinical data, revision of the manuscript; JAA carried out the collection of samples and acquisition of clinical data and participated in the revision of the manuscript; MIT carried out the collection of samples and acquisition of clinical data and participated in the revision of the manuscript; LAPJ carried out the revision of the manuscript and final approval of the version; XE carried out the conception and design of the work and participated in the revision of the manuscript and final approval of the version; MM carried out the conception and design of the work and participated in the revision of the manuscript and final approval of the version

## Supplementary Material

Additional file 1 Pedigrees of the 8 subjects with clinically relevant imbalances detected by the aCGH. (A) Case 1: 400-kb deletion at Xp11.4). (B) Case 2: 1 Mb deletion at Xp11.3. (C) Case 3: 82 kb deletion at Xq12. (D) Case 4: 800-kb duplication at Xp22.12. (E) Case 5: 700 kb duplication of at Xq28. (F) Case 6: 250 kb duplication at Xq28 (G) Case 7: 400 kb duplication at Xp11.22 (H) Case 8: 180 kb duplication at Xq12. Members tested for segregation analysis are marked with an asterisk. In all cases the imbalances segregated with pedigrees. Affected individuals are represented with black symbols. Carrier women confirmed by molecular studies are shown with a black dot inside their symbol. Click here for file

Additional file 2 Sequences of designed MLPA probes. Click here for file

Additional file 3 Primers for *OPHN1 *gene. Click here for file
