# Supplementary figures and images for: X-chromosome tiling path array detection of copy number variants in patients with chromosome X-linked mental retardation
Source: BMC Genomics. 2007 Nov 29;8:443. doi: 10.1186/1471-2164-8-443 (PMC2234261; doi:10.1186/1471-2164-8-443)

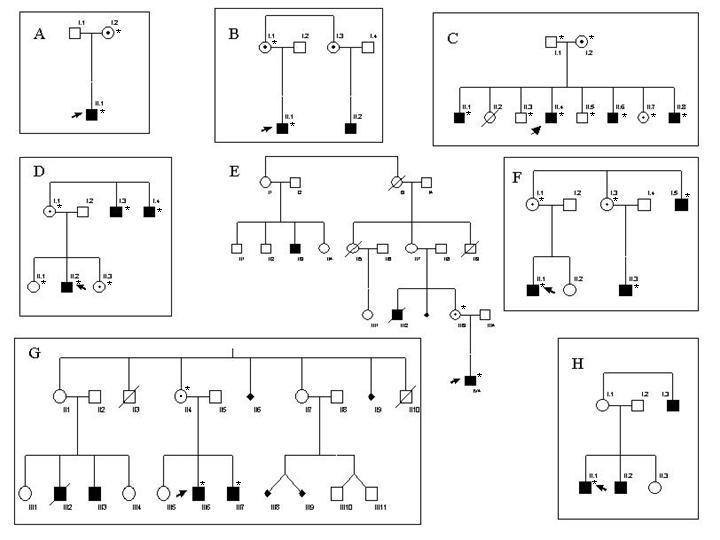

Supplement: Additional file 1 — Pedigrees of the 8 subjects with clinically relevant imbalances detected by the aCGH. (A) Case 1: 400-kb deletion at Xp11.4). (B) Case 2: 1 Mb deletion at Xp11.3. (C) Case 3: 82 kb deletion at Xq12. (D) Case 4: 800-kb duplication at Xp22.12. (E) Case 5: 700 kb duplication of at Xq28. (F) Case 6: 250 kb duplication at Xq28 (G) Case 7: 400 kb duplication at Xp11.22 (H) Case 8: 180 kb duplication at Xq12. Members tested for segregation analysis are marked with an asterisk. In all cases the imbalances segregated with pedigrees. Affected individuals are represented with black symbols. Carrier women confirmed by molecular studies are shown with a black dot inside their symbol. [file 1471-2164-8-443-S1.jpeg]
