# Supplementary material for: X-chromosome tiling path array detection of copy number variants in patients with chromosome X-linked mental retardation
Source: BMC Genomics. 2007 Nov 29;8:443. doi: 10.1186/1471-2164-8-443 (PMC2234261; doi:10.1186/1471-2164-8-443)
Supplement: Additional file 2 — Sequences of designed MLPA probes. [file 1471-2164-8-443-S2.doc]

| **Gene** | **Probe** | **Sequence** |
| --- | --- | --- |
| **HUWE1** | **L1** | GGGTTCCCTAAGGGTTGGACAGTGGAGAATATGTCATGGATGCTCG |
|  | **R1** | pTATGTGATAGGCCAGAAAGAGAGCTCTAGATTGGATCTTGCTGGCAC |
|  | **L2** | GGGTTCCCTAAGGGTTGGAGATCCAGAGAAACCAGCAGGCCAACTTG |
|  | **R2** | pGTCAGGAAGGTTCGGGAAGCTGTTGGAGTCTAGATTGGATCTTGCTGGCAC |
|  | **L3** | GGGTTCCCTAAGGGTTGGAGAGGTTCTCGCGGGATCGCGCGG |
|  | **R3** | pAGGCGGCGGTGGCTCGGTTACTGACTGCAGTAGCCGCGGATCTAGATTGGA  TCTTGCTGGCAC |
| **PHF8** | **L1** | GGGTTCCCTAAGGGTTGGAGCTTCCTTCACAGGGGGAAGAACCAACAAC |
|  | **R1** | pGCAGAGACCGTGGGAAAGAGCCCAGCCTATCTCTAGATTGGATCTTGCTGGCAC |
|  | **L2** | GGGTTCCCTAAGGGTTGGACTTCCAACAGAACGTTGGGAAGACGAG |
|  | **R2** | CAATATCTTTGGGCTGCAGAGGATCTCTAGATTGGATCTTGCTGGCAC |
| **EDA2R-** | **L1** | GGGTTCCCTAAGGGTTGGACCTTCCCACCATGGATTGCCAA |
|  | **R1** | pGAAAATGAGTACTGGGACCAATGGGGTCTAGATTGGATCTTGCTGGCAC |
|  | **L2** | GGGTTCCCTAAGGGTTGGACATGAATGCTATGTGGACAGCCCAAGCCATACCCAGAATC |
|  | **R2** | pACCTTAATTCCAACTTTTTGAGGTTCAGCAATTGTCTAGATTGGATCTTGCTGGCAC |
| **FLNA-** | **L** | GGGTTCCCTAAGGGTTGGAGCATCGAGCCCACAGGCAACATGGTGAAGA |
|  | **R** | pAGCGGGCAGAGTTCACTGTGGAGACCAGAAGTGCTCTAGATTGGATCTTGCTGGCAC |
| **EMD** | **L** | GGGTTCCCTAAGGGTTGGAGAATTCGACTAGAGGGGATGCAGATATGTATGATCTTCCCA |
|  | **R** | pAGAAAGAGGACGCTTTACTCTACCAGAGCTCTAGATTGGATCTTGCTGGCAC |

Additional file 2. Sequences of designed MLPA probes

p: phosphate (R probes are modified with a phosphate in the 5’extreme)
