# Supplementary material for: X-chromosome tiling path array detection of copy number variants in patients with chromosome X-linked mental retardation
Source: BMC Genomics. 2007 Nov 29;8:443. doi: 10.1186/1471-2164-8-443 (PMC2234261; doi:10.1186/1471-2164-8-443)
Supplement: Additional file 3 — Primers for OPHN1 gene. [file 1471-2164-8-443-S3.doc]

Additional file 3. Primers for *OPHN1* gene.

| **Gene** | **Exon** | **Primer** | **Sequence (5’->3’)** |
| --- | --- | --- | --- |
| OPHN1 | 20 | OP20F | ATCAACTGGTGGGTAGCATT |
|  |  | OP20R | GGAGCCAGGAAAGGTCTACT |
|  | 21 | OP21F | TAGTTGGTTTGTGCCACAGT |
|  |  | OP21R | CTCAGAAGGATCTCAAGGTGA |
|  | 22 | OP22F | AAGTTGGCCCAGGTAACTCT |
|  |  | OP22R | GTGATCCTGAAAGCATTCCT |
